# Bridging the computational-experimental gap: leveraging large language model to prioritize Alzheimer’s therapeutics based on comparison of learning models

**DOI:** 10.1038/s44401-026-00074-3

**Published:** 2026-02-26

**Authors:** Manqi Li, Shuteng Niu, Yifeng Xu, Jianfu Li, Xinyue Hu, Duan Liu, Merve Atik, Xiaolei Xu, Liewei Wang, Nilufer Ertekin-Taner, Cui Tao

**Affiliations:** 1 Department of Biostatistics and Data Science, School of Public Health, The University of Texas Health Science Center at Houston, Houston, TX USA; 2 McWilliams School of Biomedical Informatics, The University of Texas Health Science Center at Houston, Houston, TX USA; 3 Department of Artificial Intelligence and Informatics, Mayo Clinic, Jacksonville, FL USA; 4 Department of Electrical and Computer Engineering, University of Washington, Seattle, WA USA; 5 Department of Molecular Pharmacology and Experimental Therapeutics, Mayo Clinic, Rochester, MN USA; 6 Department of Neuroscience, Mayo Clinic, Jacksonville, FL USA; 7 Department of Biochemistry and Molecular Biology, Mayo Clinic, Rochester, MN USA; 8 Department of Cardiovascular Medicine, Mayo Clinic, Rochester, MN USA; 9 Department of Neurology, Mayo Clinic, Jacksonville, FL USA

**Keywords:** Computational biology and bioinformatics, Drug discovery, Mathematics and computing

## Abstract

Alzheimer’s Disease^1^ (AD) necessitates accelerated treatment discovery, positioning drug repurposing as a vital strategy. While computational approaches such as knowledge graph reasoning and transcriptomics show promise, they often yield divergent results, complicating the selection of candidates for experimental follow-up^2,3^. To bridge the gap between computational prediction and in vivo validation, we propose an advanced framework leveraging large language models (LLMs). We systematically evaluated three state-of-the-art computational methods (TxGNN, CompGCN, and regularized logistic regression (RLR)) to generate a unified list of 90 candidates. An LLM-based agent was then used to automate evidence synthesis from biomedical literature, mimicking expert curation to efficiently refine the list using transparent selection criteria. Validated against real-world AD patient data, clinical trial registries, and pharmacological reviews, our framework demonstrated high robustness and clinical relevance. By integrating computational predictions with scalable evidence synthesis, this approach enhances the efficiency and consistency of candidate prioritization. Ultimately, this versatile framework offers a scalable pathway to accelerate the translation of repurposed drugs for AD and other complex diseases.

## Introduction

AD is a progressive neurodegenerative disorder characterized by memory loss, cognitive decline, and behavioral disturbances, and it remains a leading cause of disability among the elderly^1^. Despite decades of research, the available therapeutic options for AD are limited in both efficacy and scope, which has driven the biomedical community to explore alternative strategies for treatment development^2,3^. One promising approach is drug repurposing, a strategy that seeks to identify new therapeutic applications for existing FDA–approved compounds^4,5^. This method offers a significant advantage by bypassing many early-phase drug development challenges, including the time and financial costs of bringing a new drug to market^2,4^. As such, drug repurposing has garnered increasing attention as a viable, pragmatic pathway for addressing the urgent need for effective AD treatments.

In recent years, advances in Artificial Intelligence (AI) and big data have reshaped the landscape of drug repurposing^6–9^. Traditional databases and expert systems cannot meet the demands of modern biomedical knowledge discovery^2,3^. As an effective solution, Knowledge graphs (KGs) have become a key data representation for high volume and complex biomedical knowledge^10–13^. Accordingly, Graph Neural Networks (GNN)-based models have been adopted to perform downstream tasks, such as KG reasoning^14,15^, transcriptomic signature analysis^16^, and integrative data mining from biomedical literature^17,18^. In particular, KG completion has emerged as a promising approach for drug repurposing for AD^14,19^. Additionally, such innovative methods have improved the quality and efficiency of drug repurposing by mining deep hidden relations in biomedical concepts without labor-based literature mining. While the SOTA GNN models have made promising progress on drug repurposing, there are several limitations: (1) the validation process remains a manual and human-centered task, resulting in poor efficiency, high costs, and a lack of scalability^19,20^, (2) different learning models show significantly varying prediction behaviors corresponding different biomedical KG characteristics^21^, and (3) the methods often lack the generalizability to be readily applied across different diseases without significant retraining.

A critical challenge in the current computational framework is the disconnect between prediction volume and validation capacity. While various advanced graph models have been proposed to address fundamental issues, such as handling sparse connections or imbalanced node types. They often exhibit significantly varying prediction behaviors based on their architectural assumptions^11^ (details and insights provided in the Discussion Model Divergence section). For instance, traditional statistical models (logistic regression, random forest) with manually-designed graph features are not suitable for complex graphs and multi-relation^22^. Another example is that regular GNN models mine deeper patterns and show better robustness to noise from KG curation, but their performance is compromised by sparse connections^23^. Moreover, GNNs equipped with node similarity matching improve node representation with sparse connections, but the drug recommendations are often less accurate in AD. Consequently, these different models generate vast, often divergent lists of candidates. This divergence exacerbates the “validation bottleneck”: traditional validation methods, which rely on expert reviews and manual literature curation, cannot scale to meet the high volume of computational predictions^24^. To date, there is no efficient and scalable way of harmonizing the outputs of different models to produce a single, reliable, and actionable list of candidates for a given disease.

To bridge this computational-experimental gap, we proposed a novel LLM-based Alzheimer’s Disease Drug Repurposing (ADDR) framework, designed to tackle the validation bottleneck by integrating the strengths of multiple graph models with the reasoning capabilities of LLMs. Our systematic approach addresses three central challenges. First, we investigated the sources of prediction divergence by conducting a comprehensive end-to-end analysis of three SOTA models (RLR, CompGCN, and TxGNN), examining their architectural principles, feature selection criteria, and pharmaceutical class representations. Second, leveraging these insights, we developed an LLM-driven module that automates prioritization by synthesizing evidence from biomedical literature, operating under strict constraints to mitigate model hallucination. Finally, we demonstrated how to efficiently verify the computational findings by corroborating our top-ranked candidates against Real-World Data (RWD), including an Electronic Health Record cohort and clinical trial data, as well as expert pharmacological reviews. In this final step, expert validation codified a reusable human-in-the-process framework for drug prioritization.

The remainder of the paper is organized as follows: Results section demonstrates key findings of the proposed framework, including prioritized and validated therapeutic candidates; Discussion section presents technical insights into model scalability, generalization capabilities, and outlines future research directions; Data sources and preparation section describes the data sources and preprocessing steps; Methods section details the model architecture and implementation; and References and Supplementary materials section includes additional experimental results, implementation details, and supporting resources.

## Results

Our unified LLM-based framework systematically prioritized 90 drug candidates generated by three SOTA graph models (TxGNN, CompGCN, and RLR). Through automated evidence synthesis of biomedical abstracts, the framework stratified these candidates into actionable categories based on literature support. Of the 90 initial predictions, the LLM identified 15 candidates as high-confidence therapeutics (Criterion 1) and 17 as having potential therapeutic relevance (Criterion 2), while 28 represented novel associations lacking current literature evidence. Subsequent validation against real-world patient cohorts, clinical trial registries, and expert pharmacological review confirmed the framework’s ability to efficiently recover established treatments (e.g., memantine) and highlight novel candidates (e.g., doxycycline) for experimental follow-up.

### LLM-prioritized therapeutic candidates

**Fig. 1:**
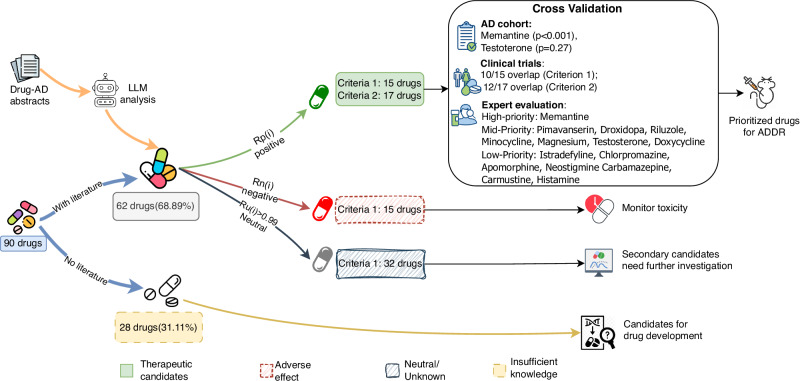
Strategy Framework of LLM-based Prioritization for AD. Ninety top-ranked drugs predicted by three graph-based models were consolidated and used to retrieve drug–Alzheimer’s disease (AD) literature from PubMed. For each drug–AD abstract, a large language model (LLM) assigned one of three evidence judgments (positive, neutral, or negative). We then aggregated these judgments into drug-level evidence scores, including a positive evidence score ${R}_{p}(i)$, a neutral evidence score ${R}_{u}(i)$, and a negative evidence score ${R}_{n}(i)$, and applied two complementary decision criteria: (i) a conservative rule that avoids forced classification when evidence is overwhelmingly neutral and otherwise selects the dominant signal, and (ii) a sensitivity-oriented rule that flags any indication of positive evidence for follow-up. The resulting stratification highlights high-confidence therapeutic candidates, drugs with potential adverse signals requiring monitoring, neutral/uncertain candidates, and drugs with insufficient published evidence. Candidates were further cross-validated using external evidence (NACC cohort analysis and overlap with AD-related ClinicalTrials.gov registrations) and expert pharmacological review to support downstream Alzheimer’s drug development prioritization. AD, Alzheimer’s disease; LLM, large language model; NACC, National Alzheimer’s Coordinating Center; ADDR, Alzheimer’s drug repurposing (framework). Symbols: ${R}_{p}(i)$, aggregated positive evidence score for drug $i$; ${R}_{u}(i)$, aggregated neutral evidence score for drug $i$; ${R}_{n}(i)$, aggregated negative evidence score for drug $i$. Color codes: light-green category indicates therapeutic candidates; red dashed-border category indicates adverse-effect signals; gray hatched category indicates neutral/unknown evidence; light-yellow category indicates insufficient knowledge.

We applied our unified LLM-based prioritization algorithm to the 90 candidate drugs identified by the three SOTA models (TxGNN^20^, CompGCN^25^, and RLR^26^, see Supplementary materials: Preliminary work) on PrimeKG^27^. After retrieving AD-relevant PubMed abstracts, we obtained a total count of judgments for each drug (${|T}_{i}|$), partitioned into positive, neutral, and negative classifications. From these counts, we computed the corresponding evidence rates (${R}_{p}$ for positive, ${R}_{u}$ for neutral, and ${R}_{n}$ for negative). Judgments were made on one abstract at a time (see Fig. 1). Notably, 28 of these 90 candidates (31.11%) yielded no AD-related publications—most frequently among TxGNN predictions (13/30), then CompGCN (8/30), and finally RLR (7/30). This lack of literature evidence highlights a critical knowledge gap and indicates that these compounds may offer novel repurposing opportunities deserving experimental validation in vitro or in vivo.

### Categorizing drug-AD relationships with LLM analysis

After excluding 28 compounds lacking AD-relevant literature, the remaining 62 candidate drugs were subjected to LLM-based evaluation using two criteria based on the statistical rates ${R}_{p}$ (positive), ${R}_{u}$ (neutral), and ${R}_{n}$ (negative). The distribution of retrieved abstracts (${T}_{i}$) for the filtered 62 candidate drugs was highly right-skewed (Supplementary Fig. S4), reflecting the uneven landscape of biomedical research. While the maximum retrieval was capped at 200 abstracts for established drugs (e.g., memantine), most candidates had sparse evidence. The median number of abstracts (${|{T|}}_{{med}}=7.5$) was significantly lower than the mean (${|{T|}}_{{mean}}=37.58,{Standard\; deviation}=63.47$), indicating that a minority of well-studied drugs dominate the literature volume and obscure candidates are far more common than well-studied ones. This uneven distribution necessitated a dual-criterion approach.

Under Criterion 1, which skipped classification when neutral responses dominate (${R}_{u}\ge 0.99$) and otherwise assigned labels based on whether ${R}_{p}$ exceeded ${R}_{n}$, 15 drugs were identified as potentially therapeutic. These included istradefylline, chlorpromazine, pimavanserin, droxidopa, and apomorphine (from TxGNN); neostigmine, carbamazepine, memantine, riluzole, minocycline, and magnesium (from CompGCN); carmustine, testosterone, doxycycline, and histamine (from RLR). Criterion 2, which adopted a more permissive rule by flagging any compound with ${R}_{p} > 0$, yielded a broader set of 17 potentially therapeutic candidates. In addition to those identified by Criterion 1, this set included haloperidol (from CompGCN) and naproxen (from RLR). These drugs constituted a prioritized shortlist for experimental validation and follow-up in the drug repurposing framework.

The evaluation further stratified compounds with ambiguous or negative literature profiles following Criterion 1. We identified 32 drugs with highly neutral sentiment distributions (${R}_{u}\ge 0.99$), reflecting either insufficient investigation or a lack of definitive conclusions in the literature. These compounds warrant targeted reviews or meta-analyses to resolve their standing in the AD therapeutic landscape.

Finally, fifteen drugs were classified as potentially adverse under Criterion 1 (${R}_{n} > {R}_{p}$ when ${R}_{u} < 0.99$), indicating a preponderance of negative evidence or safety concerns. These compounds should be deprioritized for AD repurposing and considered for further toxicological and mechanistic scrutiny prior to any future translational efforts.

### Reliability Audit and Hallucination Analysis

To strictly evaluate the risk of model hallucination^28,29^, we conducted a manual audit of 50 randomly selected abstract classifications. The LLM achieved an 82.0% agreement rate with human domain experts. Crucially, an analysis of the discordant cases (18.0%) revealed that the model’s errors were exclusively conservative. Specifically, the disagreements arose where the LLM classified abstracts containing weak or indirect evidence as “Neutral,” whereas human experts interpreted them as “Positive” based on subtle contextual cues (e.g., successful variance in preclinical biomarkers). Importantly, we observed zero instances of fabrication or “positive hallucinations”. The LLM did not classify a clearly negative or unrelated abstract as providing positive therapeutic evidence. This behavior confirms that our “context-only” prompting constraints effectively mitigate the risk of generating false leads, prioritizing high-precision validation over high-recall speculation. Detailed examples of these concordant and discordant cases are provided in Supplementary Table S5.

### Complementary contributions and distinct discovery patterns across learning models

Although the initial candidate lists generated by the three learning models were distinct, their contribution to the final prioritized set was remarkably balanced. As shown in Table 1, under the stringent Criterion 1, each model contributed approximately one-third of the high-confidence therapeutics (CompGCN: 6/15; RLR: 4/15; TxGNN: 5/15). This balance was maintained under the broader Criterion 2, with no single model dominating the results (CompGCN: 7/17; RLR: 5/17; TxGNN: 5/17).

**Table 1:** Potentially therapeutics prioritized by LLM with cross-validation

Drug Name	Graph Model	LLM-based Analysis						Hazard Ratio(p)	Clinical Trial Count (ID)
		${|T}_{i}|$	${R}_{u}(i)$	${R}_{p}(i)$	${R}_{n}(i)$	Criterion 1	Criterion 2		
Istradefylline	TxGNN	4	0.50	0.25	0.25	Potentially therapeutic	Potentially therapeutic	0.02 (0.95)	0
Chlorpromazine*	TxGNN	23	0.91	0.04	0.04	Potentially therapeutic	Potentially therapeutic	0.001 (0.93)	2(NCT02309723,NCT00975481)
Pimavanserin*	TxGNN	8	0.88	0.12	0	Potentially therapeutic	Potentially therapeutic	12.60 (0.01)	4(NCT02992132,NCT03325556,NCT03118947,NCT02035553)
Droxidopa	TxGNN	6	0.50	0.50	0	Potentially therapeutic	Potentially therapeutic	0.00 (0.90)	0
Apomorphine*	TxGNN	9	0.89	0.11	0	Potentially therapeutic	Potentially therapeutic	67.27 (<0.001)	2(NCT03837067,NCT04653584)
Neostigmine	CompGCN	5	0.80	0.20	0	Potentially therapeutic	Potentially therapeutic	<0.001 (0.92)	0
Carbamazepine	CompGCN	39	0.82	0.15	0.03	Potentially therapeutic	Potentially therapeutic	0.94 (0.82)	0
Memantine*	CompGCN	200	0.77	0.19	0.04	Potentially therapeutic	Potentially therapeutic	0.59 (<0.001)	**97(NCT00545974,NCT01626391,NCT01409694,NCT00353665,NCT05669365)**
Haloperidol	CompGCN	30	0.80	0.03	0.17	Potentially adverse effect	Potentially therapeutic	<0.001 (0.89)	4(NCT02309723,NCT00009217,NCT00000179,NCT00249145)
Riluzole*	CompGCN	7	0.71	0.29	0	Potentially therapeutic	Potentially therapeutic	1.92 (0.19)	3(NCT00353665,NCT03605667,NCT01703117)
Minocycline*	CompGCN	24	0.58	0.33	0.08	Potentially therapeutic	Potentially therapeutic	1.24 (0.57)	1(NCT01463384)
Magnesium*	CompGCN	200	0.97	0.03	0	Potentially therapeutic	Potentially therapeutic	NA	18(NCT04656860,NCT05728229,NCT00490568,NCT04606420,NCT04251182)
Carmustine	RLR	2	0.50	0.50	0	Potentially therapeutic	Potentially therapeutic	NA	0
Testosterone*	RLR	103	0.94	0.05	0.01	Potentially therapeutic	Potentially therapeutic	0.84 (0.27)	**5(NCT00392912,NCT02727699,NCT02018497,NCT00539305,NCT00000177)**
Naproxen*	RLR	37	0.76	0.05	0.19	Potentially adverse effect	Potentially therapeutic	0.94 (0.38)	4(NCT00007189,NCT01417130,NCT02702817,NCT00004845)
Doxycycline*	RLR	90	0.91	0.07	0.02	Potentially therapeutic	Potentially therapeutic	0.95 (0.81)	4(NCT00439166,NCT04846335,NCT00715858,NCT00692588)
Histamine*	RLR	61	0.98	0.02	0	Potentially therapeutic	Potentially therapeutic	NA	8(NCT01268020,NCT01505504,NCT00987220,NCT06169826,NCT01009255)

The drugs are listed in the order ranked within each graph model. Bold drugs were significant in the cohort ($p < 0.30$) and prioritized by any Criteria of LLM; “*” indicates drugs were tested in clinical trials and also prioritized by any criterion of LLM. We surveyed publicly registered trials on ClinicalTrials.gov (e.g., NCT02309723) related to the drugs predicted by our model to provide contextual evidence from prior literature. These trials were not part of our dataset or analyses, and no participants from these trials were enrolled or contacted.

Crucially, while the quantity of prioritized drugs was similar, the composition of these lists varied significantly. As detailed in Supplementary Table S1–Supplementary Table S3 and Supplementary Figure S1–Supplementary Figure S3, the models prioritized drugs from distinct pharmacological classes and chemical families, with minimal overlap between their outputs. This qualitative divergence confirms that each model mines different topological patterns within the knowledge graph. Relying on a single model would have resulted in a narrower, chemically homogenous candidate list. By integrating these diverse predictive signals, our framework successfully captured a broad spectrum of therapeutic mechanisms that no individual model could provide alone.

In summary, 62 of the 90 initial candidates were supported by AD-related literature. Of these, 15 met Criterion 1 for high-confidence therapeutic potential, 17 satisfied the broader Criterion 2, 32 were literature-neutral, and 15 showed potentially adverse signals. This clear stratification refined via LLM-based analysis, demonstrated our framework’s ability to transform diverse model outputs into a transparent and experimentally-actionable shortlist.

### Validation against real-world clinical and trial data

To test the effectiveness of our LLM-based prioritization, we first cross-validated 90 candidate compounds against an AD patient cohort (hazard ratios with p-values are shown in Table 1) and then quantified their presence in AD-related trials by investigating registered clinical trials (counts and IDs of trials are shown in Table 1).

We first evaluated our LLM-based prioritization against an AD patient cohort. As the drugs in bold (Table 1) show, among the drugs exhibiting a statistically significant protective effect, the LLM correctly recovered memantine ($p < 0.001$) as a potential therapeutic under both Criterion 1 and Criterion 2. Of four drugs with moderate cohort-level signals (zolpidem ($p=0.29$), dexamethasone ($p=0.24$), prednisolone ($p=0.14$), and testosterone ($p=0.27$)), only testosterone was prioritized under the Criteria. Remarkably, the model also flagged magnesium and carmustine as candidate therapeutics under both criteria despite no supporting evidence in the AD cohort, underscoring the LLM’s capacity to surface plausible repurposing opportunities beyond those directly observed in clinical data. While the LLM appears somewhat more conservative than cohort analysis in identifying potential associations, neither approach serves as a definitive gold standard; specifically, observational cohort findings may be confounded by unmeasured factors or limited by small sample sizes. Nonetheless, the concordance between cohort-derived associations and LLM predictions supports the utility of our LLM-prioritized framework for efficiently narrowing the search space of AD drug repurposing candidates.

Next, we assessed the 90 candidate drugs against AD-related clinical trial records (Table 1 Clinical Trial Count column). Among the candidates, twenty-eight drugs had at least one registered trial. Under Criterion 1, the LLM identified 10 of these trial-tested drugs as potential therapeutics; under Criterion 2, it recovered 12 of the 28. Using clinical trial inclusion as a proxy for prior expert endorsement, these results demonstrate that our LLM-based method can reproducibly highlight historically recognized candidates in a conservative manner, mirroring its performance in the cohort cross-validation. Moreover, of the 15 drugs flagged under Criterion 1, 10 overlapped with clinical trial compounds, leaving istradefylline, droxidopa, neostigmine, carbamazepine, and carmustine as “signal” requiring further mechanistic study. These “new” candidates illustrate the model’s ability to leverage existing biomedical knowledge and propose fresh hypotheses for future AD drug-repurposing efforts.

Taken together, the cohort and trial validations demonstrate that the LLM-based framework can recover known therapeutic signals and propose candidates overlooked by existing evidence sources.

### Expert pharmacological review for efficacy of prioritized candidates

To benchmark the LLM-based prioritization against human expertise, two independent clinicians scored 15 candidate therapeutics (those consistently highlighted by both criteria) across four domains: preclinical effectiveness, safety and tolerability, mechanism of action, and therapeutic breadth (0–4 points each, maximum 16; details were shown in Supplementary File S1). This exercise was designed to stratify LLM-prioritized agents, not to validate the framework’s global performance (no negative-control drugs were included).

Memantine ranked highest by both approaches. As shown in Table 1, it met both LLM criteria and showed significant cohort protection (${HR}=0.59,\,p < 0.001$) and the largest AD trial footprint (97 trials). Expert scores (13/16 from both reviewers; mean = 13) were concordant with this status, citing robust preclinical/clinical evidence, favorable tolerability, and a well-defined NMDA-antagonist mechanism.

Pimavanserin, droxidopa, riluzole, minocycline, magnesium, testosterone, and doxycycline were all classified as “potentially therapeutic” under LLM criteria, though trial and cohort support varied. Expert scoring placed these in the intermediate range (mean 8–9.5). Doxycycline (mean 9.5) was endorsed by both experts due to its strong preclinical effectiveness. Pimavanserin (mean 9) received solid safety ratings, consistent with LLM prioritization and its presence in four AD trials. Minocycline (mean 9) and magnesium (mean 9) were similarly reinforced by expert consensus, though magnesium lacked a cohort signal (no HR available), highlighting the LLM’s role in surfacing candidates outside limited real-world datasets. Riluzole (mean = 8.5) and droxidopa (mean = 8) both exhibited stronger preclinical efficacy than breadth of action, which the LLM captured as therapeutic potential but with modest trial representation. Testosterone (mean = 8) was captured by experts, though the AD cohort signal is weak ($p=0.27$).

Istradefylline, chlorpromazine, apomorphine, neostigmine, carbamazepine, carmustine, and histamine scored 3.5-6.5, reflecting safety concerns, limited breadth, or unclear AD-relevant mechanisms. Notably, the LLM still classified each as “potentially therapeutic”, despite a lack of clinical trial activity for most (except chlorpromazine, apomorphine, and histamine, which had 2–8 trials). This suggests that while the LLM effectively detects literature-level positive signals, expert domain knowledge remains critical for contextualizing translational feasibility and safety.

Within the LLM-selected set, expert review largely re-ordered and refined priorities rather than validating the framework in an absolute sense. Concordance at the top (e.g., memantine) and mid-tier alignment suggest that the LLM’s literature-level signals track key expert considerations, while expert adjudication adds penalties for safety liabilities and narrow mechanisms that are less evident from abstract-level text. This post-LLM triage step mirrors a human-in-the-process workflow and is readily reusable across other domains and disease areas.

## Discussion

In this study, we introduced a novel framework to bridge the persistent gap between high-throughput computational drug repurposing and practical experimental validation for AD. Our results demonstrate that by systematically reconciling the divergent outputs of multiple SOTA models and leveraging the evidence-synthesis capabilities of an LLM, we can produce a transparent, prioritized, and clinically relevant list of therapeutic candidates. This discussion will first interpret the underlying reasons for the observed model divergence, then evaluate the effectiveness, scalability, and generalizability of our proposed framework, and finally consider its current limitations and future potential.

A central challenge in computational drug repurposing is that models with comparable predictive performance often yield divergent candidate lists. Our analysis of TxGNN, CompGCN, and an RLR based on DWPC confirms this phenomenon and, more importantly, reveals that this divergence stems directly from their distinct architectures and feature representations. TxGNN emphasizes disease-similarity embeddings to produce a “low-risk” shortlist of repurposing candidates whose approved indications cluster around neurodegenerative disorders topologically proximal to AD. More than half of TxGNN’s top 30 predictions target diseases such as Parkinson’s and other nervous-system disorders (Supplementary Figure S1, Supplementary Table S1), which may expedite clinical translation by leveraging established safety profiles. The trade-off, however, lies in a potentially narrower search space that privileges well-characterized disease networks at the expense of uncovering less-explored mechanism^30^. CompGCN’s edge-composition and node-aggregation message-passing framework embeds both node and relation semantics, propagating information through densely connected subgraphs. Consequently, it uncovers a remarkably broad array of pharmacological classes, including cholinesterase modulators, antineoplastics, neuropsychiatric agents, and latent links such as memantine, despite the absence of explicit indication edges (Supplementary Figure S2, Supplementary Table S2). This diversity makes CompGCN well-suited for exploratory hypothesis generation. Yet, operating on an undirected KG without explicit semantic constraints can lead to misattribution: for example, phenobarbital, an anticholinergic with mechanisms opposed to existing cholinergic AD therapies (donepezil, rivastigmine, and galantamine)^31^, was elevated despite counter-therapeutic mechanisms. Mitigating such semantic conflation may require integrating fine-grained relation-type modeling or attention-based supervision. RLR based on DWPC transforms manually defined metapaths into interpretable graph features, favoring anti-inflammatory corticosteroids (Supplementary Figure S3, Supplementary Table S3) that align with epidemiological evidence of inflammation’s role^32^ in AD. Its transparent, interpretable design readily surfaces known drug–phenotype associations, making RLR an efficient tool for phenotype-driven candidate validation. However, its reliance on shallow, predefined four-hop metapaths limits the discovery of deeper or entirely novel pathways. These distinct profiles underscore that there is no single “best” model; instead, their strengths complement one another. The choice of model can be tailored to specific strategic goals: TxGNN for safety-oriented repurposing, CompGCN for broad mechanistic exploration, and RLR for hypothesis confirmation based on known phenotypes.

Model complementarity, however, creates a downstream prioritization problem: divergent lists expand the candidate space faster than experts can evaluate it. Our LLM-driven framework was designed specifically to address this by harmonizing heterogeneous outputs through scalable, evidence-based curation. Its effectiveness was validated through retrospective cohort analysis and cross-referencing with registered clinical trials. A distinct advantage of our “Union-then-Filter” strategy over standard ensemble methods (e.g., Borda count, rank aggregation, or probability averaging) is its ability to perform semantic rather than merely numerical validation. Standard ensembles prioritize consensus, often penalizing candidates detected by only a single model while reinforcing high-ranking errors. For instance, phenobarbital was ranked highly by CompGCN due to strong topological connectivity. A numerical ensemble would likely preserve this high rank; however, our LLM-based framework identified the context of this association as “anticholinergic” (a mechanism often contraindicated in AD) and correctly filtered it out. Conversely, our approach preserved candidates like droxidopa (uniquely identified by TxGNN) and memantine (successfully predicted by CompGCN despite the absence of an explicit indication edge in the input KG). A majority-voting system might have discarded these model-specific signals due to a lack of consensus, whereas our semantic filtering retained them for validation. This demonstrates that semantic feasibility is a superior prioritization metric to simple numerical consensus in the context of drug repurposing.

Evidence from real-world data further demonstrated that the framework can balance specificity and sensitivity. In a real-world AD cohort, the framework demonstrated a balance of specificity and sensitivity. It correctly prioritized memantine ($p < 0.001$), a drug with a robust protective signal, under both its stringent (Criterion 1) and permissive (Criterion 2) rules. For drugs with weaker cohort signals like testosterone ($p=0.27$), it selectively recovered them under the more sensitive Criterion 2, showcasing the utility of the dual-threshold design. Critically, the framework also identified candidates like magnesium, which, despite lacking a significant signal in our cohort data, was rated as a “mid-priority” compound in the subsequent expert evaluation. This illustrates the LLM’s capacity to surface plausible candidates that retrospective observational data alone might overlook.

Cross-validation against clinical trial data further confirmed the framework’s fidelity. It successfully recovered a substantial portion of previously tested drugs (6/28 under Criterion 1, 12/28 under Criterion 2), affirming its ability to reproduce expert-endorsed hypotheses. More importantly, it generated high-confidence, novel predictions such as droxidopa and carmustine, which have no prior AD trial record but were deemed plausible by our framework and, in the case of droxidopa, received a “mid-priority” expert rating. This dual capability to both confirm existing evidence and generate novel, high-potential leads underscores its value as both a validation tool and an engine for discovery.

Beyond candidate quality, our framework addresses the critical bottleneck of scalability. The retrieval and classification of 2330 PubMed abstracts for 90 candidates were completed in just over one hour, a task that would typically demand weeks of expert effort. This efficiency demonstrates that the framework can scale with a growing volume of candidates and literature without prohibitive resource demands, making rapid, literature-grounded hypothesis generation feasible across diverse therapeutic areas. Furthermore, our strict “context-only” prompting strategy enhances the framework’s utility for diseases that are less studied than AD. By constraining the LLM to reason solely from the provided text, the system functions as a rigorous gap-analysis tool. When applied to rare diseases or novel compounds where literature is sparse, the framework returns “Insufficient knowledge” or “Neutral” rather than hallucinating associations based on weak internal priors. This ensures that the prioritization reflects the actual state of scientific evidence, preventing the “self-looping” of internal model biases into the discovery process. Crucially, we pair automation with an expert-in-the-process triage step. After LLM prioritization, two independent clinicians scored the shortlisted agents to refine ordering based on translational considerations (e.g., preclinical effectiveness, safety/tolerability, mechanism, breadth). This post-LLM review mirrors a human-in-the-process framework that is easy to reuse in other domains and diseases, adding a lightweight layer of clinical judgment without re-introducing the full burden of manual curation. Together, automated literature synthesis and targeted expert adjudication deliver a scalable, generalizable platform that balances discovery breadth with translational plausibility.

Despite these strengths, several limitations warrant consideration. First, the quality and structure of the underlying KG are paramount. Sparse or undirected relationships can bias predictions, as seen with CompGCN’s mis-ranking of phenobarbital. Future work should focus on incorporating more granular, directed relation types and supervision to mitigate such semantic ambiguities. Second, regarding the graph learning component, we did not perform statistical significance testing to validate performance differences between methods. These models were selected based on their established performance in their original publications, serving as diverse “hypothesis generators” for our pipeline. As such, our focus is on harmonizing their diverse outputs to ensure a comprehensive candidate pool, rather than statistically comparing their predictive accuracy or claiming algorithmic superiority. Third, our literature analysis relies on PubMed abstracts, which may not capture the full context available in full-text articles or account for publication bias^33^. Furthermore, some novel predictions lacked any literature, highlighting a knowledge gap. To probe the reliability of our LLM’s abstract classifications, we performed a manual validation on a subset of 50 abstracts, revealing an 82.00% agreement rate with human experts. The primary sources of disagreement were twofold: 1) the LLM acted conservatively on preclinical (animal or cell) studies, often classifying them as “neutral” where an expert would see a “positive” signal, and 2) the LLM sometimes classified abstracts as “negative” when drug-AD keywords co-occurred without a stated relationship, whereas an expert would deem this “neutral”. This latter issue suggests opportunities for refinement through prompt engineering. Expanding the evidence base to include full-text articles, RWD from electronic health records, and multi-omic datasets would enhance the robustness and depth of the evidence synthesis. Fourth, a critical concern in applying LLMs to scientific validation is the risk of “data leakage,” where the model relies on its pre-trained internal knowledge rather than the provided text. To mitigate this, we employed a strict “context-only” prompting strategy (see Methods section). We qualitatively validated this constraint by observing that the model frequently classified abstracts regarding well-known AD drugs (e.g., memantine) as “Neutral” when the specific abstract text discussed non-efficacy topics, such as chemical synthesis or pharmacokinetics. This confirms that the framework adheres to the context window and avoids hallucinating therapeutic signals based on prior training data. While effective, future iterations could be further enhanced by fine-tuning models on domain-specific biomedical literature to improve nuance in interpreting preclinical results. Fifth, the uneven availability of literature introduces a “popularity bias” into the prioritization scores. As shown in our Results section and Supplementary Figure S4, the distribution of ${{|T}}_{i}|$ is heavily skewed. This disparity affects the statistical interpretation of the classification rates (${R}_{p}$, ${R}_{u}$, ${R}_{n}$). Drugs with high ${{|T}}_{i}|$ (e.g., memantine, ${{|T}}_{i}| > 200$) benefit from statistical robustness; for these drugs, ${R}_{p}$ represents a consensus derived from hundreds of data points, making the LLM’s output highly reliable. In contrast, for novel candidates with low ${{|T}}_{i}|$ (e.g., ${{|T}}_{i}| < 5$), the rates are subject to high variance, where a single positive abstract can shift ${R}_{p}$ from 0.00 to 0.50. Crucially, this variance is not a failure of the model but a reflection of the “novelty” of the candidate. A strict statistical threshold (like Criterion 1) penalizes these low-evidence drugs. To mitigate this, our Criterion 2 was explicitly designed as a high-sensitivity filter. By prioritizing candidates with any positive signal (${R}_{p} > 0$), we ensure that “low-volume, high-potential” candidates are flagged for experimental validation rather than being discarded due to a lack of historical publication volume.

Looking forward, we envision evolving this linear framework into a dynamic, closed-loop discovery ecosystem. While the current “human-in-the-process” design uses expert review as a final validation step, future iterations will feed these expert scores back into the pipeline to fine-tune the graph embeddings and LLM prompts. A virtuous cycle could be established where the top in-silico predictions are validated in preclinical models (e.g., organoids) and reviewed by clinicians, with the resulting data serving as ground-truth labels to retrain the models. This iterative approach would progressively enhance predictive accuracy and accelerate the translation of computational insights into tangible clinical impact.

Overall, computational drug repurposing for complex illnesses like Alzheimer’s Disease is often hampered by predictive models that yield divergent, overwhelming candidate lists, creating a significant bottleneck for experimental validation and clinical translation. In this work, we have demonstrated a novel hybrid framework that successfully overcomes this challenge. By integrating the outputs of complementary graph-based algorithms and leveraging a Large Language Model for rapid, automated evidence synthesis, we transformed a wide array of computational predictions into a single, prioritized list of therapeutic candidates. Our approach was validated through real-world patient data, clinical trial records, and expert pharmacological review, confirming its ability to identify both established treatments and promising new candidates. The framework’s true strength lies in its capacity to harmonize high-throughput in silico screening with deep, contextualized evidence appraisal in a manner that is transparent, scalable, and reproducible. This methodology not only accelerates the discovery of viable therapeutics for AD but also provides a powerful and generalizable blueprint for bridging the critical gap between computational prediction and clinical application across a spectrum of human diseases.

## Methods

**Fig. 2:**
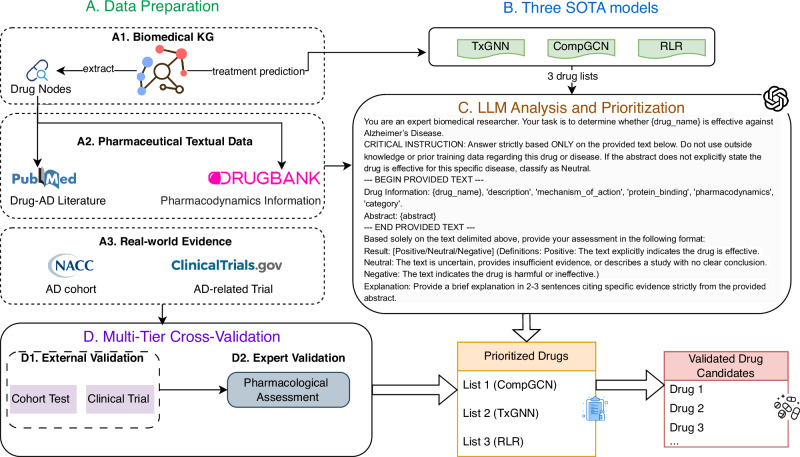
Framework Graph of LLM-based Prioritization for AD Therapeutics. Blocks **A**–**D** summarize the full prioritization pipeline. **A** Data preparation: drug nodes and drug–disease relations are extracted from a biomedical knowledge graph (PrimeKG), and complementary textual evidence is assembled from PubMed drug–AD literature and DrugBank pharmacology fields; real-world and prospective evidence sources include the NACC AD cohort and AD-related registrations on ClinicalTrials.gov. **B** Candidate generation: three state-of-the-art (SOTA) models (TxGNN, CompGCN, and a regularized logistic regression model based on degree-weighted path counts, RLR) produce three ranked drug lists. **C** LLM analysis and prioritization: an LLM applies a constrained prompt to the provided drug information and abstract text to label evidence as Positive/Neutral/Negative and generate a prioritized list. **D** Multi-tier cross-validation: prioritized drugs are evaluated using external validation (cohort-based testing and clinical-trial evidence) and expert validation (pharmacological assessment) to yield a final set of validated candidates. AD, Alzheimer’s disease; KG, knowledge graph; SOTA, state-of-the-art; LLM, large language model; NACC, National Alzheimer’s Coordinating Center; CompGCN, compositional graph convolutional network; RLR, regularized logistic regression. Color codes: the stage headers use distinct colors to denote blocks **A**–**D**; dashed-outline boxes indicate submodules within a stage (e.g., external vs expert validation).

The methods for this study are organized into four principal stages: data preparation, candidate generation, LLM-driven prioritization, and multi-tier validation. A detailed schematic of this framework is presented in Fig. 2, which outlines the flow from data aggregation to the final validated drug candidates.

### Problem definition

The central problem this research addresses is the systematic prioritization of existing drugs for repurposing as AD therapeutics. This involves bridging the gap between divergent computational predictions and actionable experimental validation. We formulate this as a multi-stage process involving knowledge graph-based prediction, LLM-driven evidence synthesis, and multi-faceted evaluation.

Let $G=(V,\varepsilon )$ be a biomedical KG whose nodes include drug nodes ${V}_{{drug}}\subseteq V$ and disease nodes ${V}_{{disease}}\subseteq V$. Given a target disease ${d}^{* }\in {V}_{{disease}}$ and a biomedical corpus ${T=\{({{dr}}_{i},{T}_{i})\}}_{i=1}^{n}$, where ${{dr}}_{i}\in {V}_{{drug}}$ is a candidate drug and ${T}_{i}$ is the set of textual evidence for drug candidates ${{dr}}_{i}$, the objective is to generate a prioritized and stratified list of drug candidates for treating ${d}^{* }$. We employ $m$ graph models that predict the probability that each ${{dr}}_{i}$ treats ${d}^{* }$:

1 $${f}_{j}({{dr}}_{i}|{d}^{* },G)\in [0,1],j=1,...,m$$

For each model, let ${L}_{j}$ denote the top-ranked predictions under a threshold. The candidate pool is the union:

2 $$L={\cup }_{j=1}^{m}{L}_{j}$$

To aggregate textual evidence, an LLM–based labeling function, $\phi ({d}^{* },{T}_{i})\in \{+\mathrm{1,0},-1\}$ maps each drug’s textual evidence ${T}_{i}$ to a relevance label (positive, neutral, negative) with respect to ${d}^{* }$. A universal prioritization function $\psi ({{dr}}_{i},{f}_{j}({{dr}}_{i}|{d}^{* },G),\phi ({d}^{* },{T}_{i}))\in \{\mathrm{0,1}\}$ determines whether ${{dr}}_{i}$ should be included in the prioritized list. The final output is a stratified collection of validated drug candidates:

3 $$\widehat{D({d}^{* })}=\{{{dr}}_{i}\in {L|}\psi ({{dr}}_{i},\cdot ,\cdot )=1,{V}_{{ext}}({{dr}}_{i},{d}^{* })=1,{V}_{\exp }({{dr}}_{i},{d}^{* })=1\}$$

where ${V}_{{ext}}$ and ${V}_{\exp }$ denote validation functions using external evidence (e.g., medical cohort for ${d}^{* }$, trials) and expert assessment, respectively.

### Biomedical Knowledge Graph Selection and Features

PrimeKG is a comprehensive biomedical KG comprising 129,375 nodes across 10 entity types and 4,050,249 edges across 33 relation types^27^. It integrates data from 20 publicly available resources to enable rich and multi-domain connectivity, including DisGeNET, DrugBank, SIDER, Gene Ontology, Reactome, UBERON, etc. Critically, PrimeKG serves as a harmonized dataset where extensive entity resolution and standardization have been performed upstream. This curation process ensures data quality by resolving duplicates and mapping drug entities to standardized vocabularies (e.g., DrugBank, Drug Central), thereby eliminating the need for the additional raw data preprocessing. Compared with existing networks such as Hetionet^34^, iBKH^35^, and PharmKG^36^, PrimeKG is particularly adept at reasoning about repurposable drugs due to its significantly more extensive drug-drug relation(33.0%). PrimeKG categorizes drug-disease relations into three types: “Drug_contraindication_Disease”, “Drug_indication_Disease”, and “Drug_off-label use_Disease”. This unique classification facilitates machine learning and deep learning efforts to explore drug-disease connectivity with different semantic meanings. Our study focuses on predicting the “indication” relation between AD and each of the 7957 drug nodes in PrimeKG.

### Complementary pharmaceutical corpora for LLM analysis

To leverage the summarization and classification capabilities of LLM, we assembled two complementary corpus, pharmacological descriptions from DrugBank (https://go.drugbank.com^37^) and drug–AD abstracts from PubMed^38^. DrugBank has served as a gold-standard repository of drug information since 2006, offering detailed annotations of drug targets, pharmacodynamics, and chemical categories. For each of the 7957 PrimeKG drug entities, we extracted the “Description”, “Pharmacology” (indication, pharmacodynamics, mechanism of action, protein binding), and “Category” fields. This textual metadata enriches the purely structural information in PrimeKG, mitigating the limitations of graph-only representations. PubMed (https://pubmed.ncbi.nlm.nih.gov/about/) provides access to MEDLINE^39^ and contains over 38 million biomedical citations. We queried PubMed using the names and synonyms (from DrugBank and MeSH) of the top 90 candidates predicted by three ADDR models, in conjunction with “Alzheimer Disease”. Limiting retrieval to abstracts published from 2000 onward and capping at 200 abstracts per query, we collected a total of 2330 abstracts. These texts supply context on reported drug–AD associations and serve as input for the LLM’s evidence-synthesis framework.

### External validation resource

We leveraged both Alzheimer’s cohorts dataset and clinical-trial registries to externally validate our LLM-based prioritization. First, we obtained the Uniform Data Set (UDS) from the National Alzheimer’s Coordinating Center (NACC^40^), one of the largest, longest-running Alzheimer’s cohorts, on June 17, 2024. The dataset comprises 188,701 longitudinal visit records through the March 2024 data freeze. These data are de-identified and were obtained from NACC via its standard request process and under its data use agreement; there was no interaction with human participants and no access to identifiable private information. The research involved only secondary data analysis; accordingly, informed consent and continuing review were not required, and the requirement for informed consent was waived as determined by the approving ethics committees. Ethics oversight and determinations were provided by the Mayo Clinic Institutional Review Board (IRB) (IRB# 24-000917, IRB title: “Artificial intelligence and informatics study for clinical decision support, risk prediction, and drug repurposing”; exempt from continuing review on February 26, 2024) and the University of Texas Health Science Center at Houston Committee for the Protection of Human Subjects (IRB# HSC-SBMI-21-0965; IRB title: “Deep learning and knowledge graph on EHR for clinical decision support, risk prediction, and drug-repurposing”; expedited review and approval on November 15, 2021). All procedures adhered to the principles of the Declaration of Helsinki. We fitted a left-truncated, right-censored Cox proportional hazards model to investigate drug efficacy (see section Methods: Statistical Analysis of the NACC Alzheimer’s Cohorts).

Second, we queried ClinicalTrials.gov (https://clinicaltrials.gov/) for AD–related drug trials and cross-referenced these against our LLM-prioritized list. NCT identifiers (e.g., NCT02309723) cited in this article refer to independent, external clinical trials not conducted by the authors and are included solely as supporting literature to contextualize our drug-repurposing predictions**;** no trial data or participants from these external studies were enrolled, contacted, or analyzed in this work. By triangulating real-world outcomes from NACC with prospective evidence from registered trials, this multi-faceted approach demonstrates the robustness of our LLM validation and highlights candidates already evaluated in human studies for further investigation.

### Candidate Generation Models

To generate the initial candidate pool, we employed three SOTA computational models: TxGNN, CompGCN, and RLR based on DWPC. Each of these models demonstrated exceptional performance on its original benchmark: TxGNN achieved an AUPRC of 0.913, CompGCN an MRR of 0.355, and the RLR model an AUROC of 0.974. Although originally evaluated on different datasets and metrics, we applied them uniformly to the PrimeKG knowledge graph to predict AD indications and generate candidate drug lists for LLM-based prioritization. To ensure a fair union, we aligned the evaluation logic across all models under a Closed World Assumption, defining the negative set for prioritization as {All Drug-Disease Pairs} - {Known Indications}. The search space comprised all 7957 drug nodes in PrimeKG. To strictly evaluate repurposing potential, the 8 known FDA-approved AD therapeutics (epicriptine, pramiracetam, acetylcarnitine, ipidacrine, galantamine, tacrine, rivastigmine, and donepezil) were masked during the ranking phase. TxGNN was evaluated using its pre-trained weights and fine-tuned scores (zero-shot setting). CompGCN and RLR were retrained specifically on PrimeKG for our study. Below we detail the architecture, model training, negative sampling strategies, and ranking criteria.

TxGNN is a graph model designed for zero-shot drug repurposing, enabling the identification of potential therapeutic candidates even for diseases lacking established treatments. It employs a Graph Neural Network (GNN), a model that iteratively updates node representations by aggregating information from their neighbors, within a large-scale medical knowledge graph (PrimeKG). One of the cores about TxGNN is that it generates vector embeddings for drugs${(e}_{{drug}})$ and diseases ${(e}_{{disease}})$ based on topological similarity.

The predictive model can be conceptualized as

4 $$y \sim {f}_{{TxGNN}}({e}_{{drug}},{e}_{{disease}})$$

In this formula, the outcome $y$ is a predicted score indicating the likelihood that the drug is an indication for the disease. The predictor ${(e}_{{drug}},{e}_{{disease}})$ are the learned vector embedding of the drug and disease. These embeddings are intermediate representations generated by the GNN encoder. Specifically, ${e}_{{drug}}$ represents the learned latent vector embedding for the specific drug being queried. This embedding captures information about how the drug connects to targets within one hop, associated pathways, known indications/contraindications, genes, etc.

Similarly, ${e}_{{disease}}$ represents the learned latent vector embedding for the specific disease being queried, which captures information about how the disease connects to targets within one hop.

We utilized the official “off-the-shelf” results provided by the original authors. We extracted the fine-tuned prediction scores generated by the pre-trained TxGNN model on the PrimeKG dataset. This preserves the exact zero-shot evaluation setting reported in the original publication. As detailed in the original publication, TxGNN employs a two-stage process. The first stage is pretraining, which uses random negative sampling (1:1 ratio) to initialize embeddings. The second stage is finetuning, which utilizes a full negative sampling (1-vs-All) strategy within a metric learning module. For each disease in the support set, the model is trained to rank true therapeutic candidates higher than all other candidate drugs in the graph. Rankings for TxGNN (Table 1 and Supplementary Table S1) are based on the fine-tuned similarity scores from the metric learning decoder.

CompGCN is a framework designed to learn representations from multi-relational graphs, where nodes are connected by edges that have specific types (relations) and directions. Unlike traditional Graph Convolutional Networks (GCNs) that primarily handle simple, undirected graphs or struggle with the complexity and parameter explosion of numerous relation types, CompGCN learns vector embeddings for both the nodes (entities) and the relations simultaneously. It achieves this by incorporating composition operations (e.g., subtraction, multiplication, circular-correlation) inspired by KG embedding techniques. During the message passing phase, the representation for a target node is updated by aggregating information from its neighbors, where each neighbor’s contribution is computed by composing the neighbor node’s embedding with the embedding of the connecting relation using a chosen operator.

To manage the complexity and potential over-parameterization, CompGCN uses relation-type specific weight matrices (e.g., distinct weights for incoming, outgoing, and self-loop edges) and transforms relation embeddings alongside node embeddings across layers. This allows relation information to be shared and refined throughout the network. Furthermore, it offers a scalable approach for graphs with many relations by optionally using a basis decomposition technique, where initial relation embeddings are represented as linear combinations of a smaller, learnable set of basis vectors. This method generalizes several prior multi-relational GCN approaches and is shown to be effective for tasks requiring understanding of graph structure and relationships, such as link prediction.

To apply the CompGCN to predict treatment relation between drugs and diseases, we frame the prediction model as follow:

5 $$y \sim {f}_{{CompGCN}}({h}_{{drug}},{{h}_{{relation}},h}_{{disease}})$$

where $y$ represents the score indicating the existence of a specific relation between a particular drug and a particular disease. The predictors ${(h}_{{drug}},{h}_{{relation}},{h}_{{disease}})$ are the features derived from the CompGCN’s learned embeddings for specific drug, relation and disease. The model essentially generates these predictors as input features to get a scoring function ${f}_{{CompGCN}}$ to produce the outcome $y$. We utilized the ConvE score function and circular-correlation composition operation due to their preliminary performance.

We retrained CompGCN on PrimeKG specifically for this study. We utilized a standard 1-vs-All training strategy. For every positive drug-disease triplet in a training batch, the model scores it against all other possible disease entities simultaneously. The model optimizes a binary cross entropy loss where all unobserved triplets are treated as negatives. Consistent with our Closed World Assumption for AD repurposing, any pair lacking an explicit “Indication” edge is treated as a negative sample. Rankings of CompGCN (Table 1 and Supplementary Table S2) are based on the output prediction score of the re-trained link predictor.

RLR was developed in the Hetionet project (https://github.com/dhimmel/learn) to capture the meta-path topology of any drug–disease pair in the KG and utilizes network topology as its primary feature set. Specifically, RLR employs Degree-Weighted Path Counts (DWPC), a metric designed to quantify the prevalence of specific path types between a source and target node while down-weighting highly connected, less informative nodes.

A meta-path ($\lambda$) is a schema (e.g. Drug→Gene→Phenotype→Disease) describing a sequence of node types and edge types. For each meta-path $\lambda$, the DWPC between a source node $s$ and target node $t$ is defined as the sum over all path instances $p$ of the product of each intermediate node’s degree raised to the negative damping exponent $-w=-0.4$:

6 $${{DWPC}}_{\lambda }\left(s,t\right)=\mathop{\sum }\limits_{p\in {P}_{\lambda }(s,t)}\left\{\mathop{\prod }\limits_{v\in p{\rm{\backslash }}\{s,t\}}{deg(v)}^{-w}\right\}$$

Here, ${P}_{\lambda }(s,t)$ is the set of all walks of type $\lambda$ from $s$ to $t$, $deg(v)$ is the degree of each intermediate node $v$, and $w$ controls how strongly high-degree nodes are down-weighted.

This formulation ensures that having many connecting walks increases the feature value, while walks through highly connected (less informative) nodes are down-weighted. Each meta-path’s DWPC then serves as a feature in an elastic-net-regularized logistic regression (via the glmnet package) that predicts the probability of a therapeutic relationship between the source drug and target disease; regularization both prevents overfitting across hundreds of meta-path features and induces sparsity in the learned coefficients. An RLR is trained on these DWPC features to predict the probability y of a therapeutic relationship:

7 $${Logit}\left(y\right)={\beta }_{0}+\mathop{\sum }\limits_{\lambda }{\beta }_{\lambda }{DWP}{C}_{\lambda }(s,t)$$

Here ${\beta }_{0}$ is the intercept, and ${\beta }_{\lambda }$ represents the coefficient for the meta path $\lambda$ from source s to target t. In addition to DWPC features, the model incorporates a prior probability of indication derived from node-degree–based prevalence.

We retrained the RLR model on PrimeKG using the glmnet package with elastic-net regularization to handle feature correlation and induce sparsity. Because the original Cypher-based feature extraction relied on network permutations and proved computationally intensive, we utilized the hetnet_ml framework (https://github.com/mmayers12/hetnet_ml) to accelerate DWPC computation in our treatment prediction on PrimeKG. We operated RLR under the Closed World Assumption. The positive class ($y=1$) consisted of existing “Indication” edges. The negative class ($y=0$) was defined as all drug-disease pairs lacking an indication edge in the graph. We utilized the full set of non-indications to ensure the model learned global network topology. Rankings for RLR (Table 1 and Supplementary Table S3) are based on the predicted probabilities from the logistic regression classifier.

### ADDR Framework Implementation

The LLM-based framework was executed within the Google Colaboratory environment on a virtual machine equipped with an NVIDIA T4 GPU and 16 GB of GDDR6 memory. The analysis of all PubMed abstracts was completed in approximately 1 hour and 11 min. To generate an initial candidate set, we first evaluated three SOTA models on the PrimeKG knowledge graph for AD indication prediction. We extracted the top 30 ranked candidates from each model. This cutoff was selected to ensure a manageable yet diverse pool for high-depth evidence synthesis, aligning with standard screening capacities for downstream experimental validation (typically 20–50 candidates^13,19^). This yielded a consolidated set of 90 unique drugs.

For each drug–AD pair, we retrieved the relevant abstracts and utilized a structured prompt (see Fig. 2) for an LLM (GPT4.1 API). We specifically selected GPT-4.1 over domain-specific foundation models such as BioGPT^41^ or ProtBERT^42^ for this component. While BERT-based biomedical models excel at entity recognition and sequence embedding, our framework requires high-level zero-shot reasoning to synthesize unstructured arguments (e.g., distinguishing between a drug being merely “mentioned” versus showing “therapeutic efficacy”). GPT-4.1’s superior capabilities in semantic generalization and summarization make it uniquely suitable for this complex evidence harmonization task^43^.

We tasked the model to classify the relationship between drug and AD in each abstract as “positive”, “neutral”, or “negative”. For each drug ${d}_{i}$, the LLM produced a set of judgements from a total of $|{T}_{i}|$ abstracts, which can be partitioned into positive (${P}_{i}$), neutral (${U}_{i}$), and negative (${N}_{i}$). From these, we defined three rates to quantify the drug’s overall evidence profile:

$${R}_{p}(i)=\frac{{P}_{i}}{|{T}_{i}|},{R}_{u}(i)=\frac{{U}_{i}}{|{T}_{i}|},{R}_{n}(i)=\frac{{N}_{i}}{|{T}_{i}|}$$

where $|{T}_{i}|={P}_{i}+{U}_{i}+{N}_{i}$ and consequently ${R}_{p}(i){+R}_{u}(i){+R}_{n}(i)=1$. These rates form the basis of our unified ranking algorithm, ensuring each candidate is assessed by the same quantitative criterion.

Two distinct criteria were defined to accommodate different decision-making needs. Criterion 1 was designed to identify candidates with a clear signal by first filtering out those with overwhelmingly inconclusive evidence. Criterion 2 was designed to maximize sensitivity, flagging any candidate with even a single piece of positive evidence. Formally, for each drug ${d}_{i}$ :

$${{Criterion}}_{1}(i)=\left\{\begin{array}{c}{Neutral}\,{Relation}\,{R}_{u}(i)\ge 0.99,\\ {Potentially}\,{Therapeutic}\,{R}_{u}(i) < 0.99\,\wedge {\,R}_{p}(i)\ge \,{R}_{n}(i),\\ \,{Potentially}\,{Adverse}\,{Effect}\,{R}_{u}\left(i\right) < 0.99\,\wedge {\,R}_{n}\left(i\right)\ge {R}_{p}\left(i\right),\end{array}\right.$$

$${{Criterion}}_{2}(i)=\left\{\begin{array}{l}{Potentially}\,{Therapeutic}{\,\,\,\,R}_{p}(i) > 0,\\ {No}\,{Positive}\,{Sign}\,\,\,\,\,\,\,\,\,\,\,\,\,\,\,{otherwise}.\end{array}\right.$$

Together, Criterion 1 captures nuanced, context-dependent judgments while Criterion 2 ensures that no candidate with even minimal positive evidence is overlooked.

### Statistical analysis of the NACC Alzheimer’s cohorts

The UDS from the NACC^40^ comprised 188,701 longitudinal visits from 50,962 participants. At baseline, the mean age was 71.18 ± 10.41(mean ± SD) years and mean years of education were 15.85 ± 7.96 (mean ± SD); 57.19% were female and 42.81% male. At enrollment, marital status was distributed as 63.20% married, 16.06% widowed, 11.68% divorced, and the remainder separated, never married, cohabiting/domestic partners, or other. The living situation at enrollment was 61.63% with spouse/partner, 23.12% alone, 9.05% with a relative or friend, 4.00% in group residence, and the rest other or unknown. Cognitive status at enrollment was 40.39% normal cognition, 4.43% impaired-not-MCI, 22.30% MCI, and 32.88% dementia; at the end of follow‑up, these proportions were 41.39%, 4.09%, 18.29%, and 36.22%, respectively. Drug exposures in the UDS were mapped to PrimeKG drugs via UMLS and Drugbank identifiers. To approximate chronic use, only drugs documented at two or more consecutive visits were included in the analysis.

To estimate each drug’s association with the time to cognitive decline, we fitted a Cox proportional hazards model. This semi-parametric model is ideally suited for this analysis as it accommodates time-to-event data characterized by left-truncation (participants entering the study at different ages) and right-censoring (participants leaving the study for reasons other than the event of interest, such as death or loss to follow-up), both of which are features of the NACC longitudinal cohort^44^. The model was defined as:

8 $$\begin{array}{l}h(t|{X}_{i})={h}_{0}(t)\exp ({\beta }_{drug}{X}_{i,drug}+{\beta }_{age}{X}_{i,age}+{\beta }_{sex}{X}_{i,sex}+{\beta }_{edu}{X}_{i,edu}\\ +{\beta }_{mar}{X}_{i,mar}+{\beta }_{live}{X}_{i,live})\end{array}\,$$

where ${h}_{0}(t)$ is the baseline hazard function, the covariates ${X}_{i}$ included the binary indicator for chronic drug use as well as baseline age, sex, education, marital status, and living situation for participant $i$, and each $\beta$ is the log-hazard coefficient for its covariate. Our analysis focused on the hazard ratio for the drug effect, ${{HR}}_{{drug}}=\exp ({\beta }_{{drug}})$, which quantifies the relative hazard of cognitive decline for exposed versus non-exposed participants. An ${{HR}}_{{drug}} > 1$ indicates an increased risk (adverse effect), while an ${{HR}}_{{drug}} < 1$ suggests a protective effect.

Statistical significance was evaluated at two distinct thresholds. We used the conventional alpha level of $p < 0.05$ to declare a statistically significant association, as this standard minimizes the probability of a Type I error (a false positive^45^). In addition, we considered a more relaxed threshold of $p < 0.30$ for exploratory purposes, as noted in Table 1. This less stringent cutoff is often used in epidemiological and discovery-oriented research to identify potentially meaningful trends or signals that might be missed with a stricter threshold, thereby reducing the risk of Type II errors (false negatives) at the cost of including some false positives in a preliminary screening phase^46,47^.

## Supplementary information

Supplementary information

## Data Availability

Data from NACC can be requested through https://naccdata.org/requesting-data/submit-data-request. Data from PrimeKG are available at https://dataverse.harvard.edu/dataset.xhtml?persistentId=doi:10.7910/DVN/IXA7BM. Other datasets generated and analyzed during the current study are available at https://github.com/maggielee1111/LLM-prioritization-Framework.

## Code availability

The underlying code and datasets for this study are available via this link https://github.com/maggielee1111/LLM-prioritization-Framework.

## References

[1] 2023 Alzheimer’s disease facts and figures. *Alzheimers Dement*. **19**, 1598–1695 (2023). 10.1002/alz.13016 36918389

[2] Tanoli, Z. et al. Computational drug repurposing: approaches, evaluation of in silico resources and case studies. *Nat. Rev. Drug Discov.* **24**, 521–542 (2025). 40102635 10.1038/s41573-025-01164-x

[3] Pushpakom, S. et al. Drug repurposing: progress, challenges and recommendations. *Nat. Rev. Drug Discov.* **18**, 41–58 (2019). 30310233 10.1038/nrd.2018.168

[4] Cummings, J., Lee, G., Zhong, K., Fonseca, J. & Taghva, K. Alzheimer’s disease drug development pipeline: 2021. *Alzheimers Dement. Transl. Res. Clin. Interv.* **7**, e12179 (2021). 10.1002/trc2.12179 PMC8145448 34095440

[5] Ianevski, A. et al. RepurposeDrugs: an interactive web-portal and predictive platform for repurposing mono- and combination therapies. *Brief. Bioinform.* **25**, bbae328 (2024). 38980370 10.1093/bib/bbae328 PMC11232279

[6] Yan, C. et al. Leveraging generative AI to prioritize drug repurposing candidates for Alzheimer’s disease with real-world clinical validation. *Npj Digit. Med.* **7**, 46 (2024). 38409350 10.1038/s41746-024-01038-3 PMC10897392

[7] Li, Y. et al. RefAI: a GPT-powered retrieval-augmented generative tool for biomedical literature recommendation and summarization. *J. Am. Med. Inform. Assoc*. ocae129 10.1093/jamia/ocae129 (2024). 10.1093/jamia/ocae129 PMC11339508 38857454

[8] Aldahdooh, J., Vähä-Koskela, M., Tang, J. & Tanoli, Z. Using BERT to identify drug-target interactions from whole PubMed. *BMC Bioinformatics* **23**, 245 (2022). 35729494 10.1186/s12859-022-04768-x PMC9214985

[9] Aldahdooh, J., Tanoli, Z. & Tang, J. Mining drug–target interactions from biomedical literature using chemical and gene descriptions-based ensemble transformer model. *Bioinforma. Adv.* **4**, vbae106 (2024). 10.1093/bioadv/vbae106 PMC11293871 39092007

[10] Nicholson, D. N. & Greene, C. S. Constructing knowledge graphs and their biomedical applications. *Comput. Struct. Biotechnol. J.* **18**, 1414–1428 (2020). 32637040 10.1016/j.csbj.2020.05.017 PMC7327409

[11] Kumar, A. A., Bhandary, S., Hegde, S. G. & Chatterjee, J. Knowledge graph applications and multi-relation learning for drug repurposing: A scoping review. *Comput. Biol. Chem.* **115**, 108364 (2025). 39914071 10.1016/j.compbiolchem.2025.108364

[12] Su, C. et al. CBKH: The Cornell Biomedical Knowledge Hub.

[13] Al-Saleem, J. et al. Knowledge graph-based approaches to drug repurposing for COVID-19. *J. Chem. Inf. Model.* **61**, 4058–4067 (2021). 34297570 10.1021/acs.jcim.1c00642

[14] Hu, X. et al. Self-Explainable Graph Neural Network for Alzheimer Disease and Related Dementias Risk Prediction: Algorithm Development and Validation Study. *JMIR Aging* **7**, e54748 (2024). 10.2196/54748 PMC11263893 38976869

[15] Liu, Y., Huse, J. & Kannan, K. Expression graph network framework for biomarker discovery. Brief. Bioinform. 26, bbaf559 (2025). 10.1093/bib/bbaf559 PMC12554635 41139924

[16] Li, G. et al. Transcriptomic signatures and repurposing drugs for COVID-19 patients: findings of bioinformatics analyses. *Comput. Struct. Biotechnol. J.* **19**, 1–15 (2021). 33312453 10.1016/j.csbj.2020.11.056 PMC7719282

[17] Duong Nguyen, T. T. et al. PGxDB: an interactive web-platform for pharmacogenomics research. *Nucleic Acids Res* **53**, D1486–D1497 (2025). 39565203 10.1093/nar/gkae1127 PMC11701576

[18] Wang, Y. et al. DrugRepo: a novel approach to repurposing drugs based on chemical and genomic features. *Sci. Rep.* **12**, 21116 (2022). 36477604 10.1038/s41598-022-24980-2 PMC9729186

[19] Nian, Y. et al. Mining on Alzheimer’s diseases related knowledge graph to identity potential AD-related semantic triples for drug repurposing. *BMC Bioinformatics* **23**, 407 (2022). 36180861 10.1186/s12859-022-04934-1 PMC9523633

[20] Huang, K. et al. A foundation model for clinician-centered drug repurposing. *Nat. Med.* **30**, 3601–3613 (2024). 39322717 10.1038/s41591-024-03233-x PMC11645266

[21] Qiu, Y., Zhang, Y., Deng, Y., Liu, S. & Zhang, W. A comprehensive review of computational methods for drug-drug interaction detection. *IEEE/ACM Trans. Comput. Biol. Bioinform.* **19**, 1968–1985 (2022). 34003753 10.1109/TCBB.2021.3081268

[22] Jaeger, M. Learning and reasoning with graph data. *Front. Artif. Intell.* **6**, 1124718 (2023). 37675398 10.3389/frai.2023.1124718 PMC10477700

[23] Wu, Z. et al. A comprehensive survey on graph neural networks. *IEEE Trans. Neural Netw. Learn. Syst.* **32**, 4–24 (2021). 32217482 10.1109/TNNLS.2020.2978386

[24] Pillai, M. & Wu, D. Validation approaches for computational drug repurposing: a review. *AMIA Annu. Symp. Proc. AMIA Symp* **2023**, 559–568 (2023). 38222367 PMC10785886

[25] Vashishth, S., Sanyal, S., Nitin, V. & Talukdar, P. Composition-based multi-relational graph convolutional networks. *ICLR Conference* (2020).

[26] Himmelstein, D. S. et al. Systematic integration of biomedical knowledge prioritizes drugs for repurposing. *eLife* **6**, e26726 (2017). 28936969 10.7554/eLife.26726 PMC5640425

[27] Chandak, P. PrimeKG. Harvard Dataverse 10.7910/DVN/IXA7BM (2022).

[28] Huang, L. et al. A survey on hallucination in large language models: principles, taxonomy, challenges, and open questions. *ACM Trans. Inf. Syst.* **43**, 1–55 (2025).

[29] Metze, K., Morandin-Reis, R. C., Lorand-Metze, I. & Florindo, J. B. Bibliographic research with ChatGPT may be misleading: the problem of hallucination. *J. Pediatr. Surg.* **59**, 158 (2024). 37735041 10.1016/j.jpedsurg.2023.08.018

[30] Truong, T. T. T., Panizzutti, B., Kim, J. H. & Walder, K. Repurposing drugs via network analysis: opportunities for psychiatric disorders. *Pharmaceutics* **14**, 1464 (2022). 35890359 10.3390/pharmaceutics14071464 PMC9319329

[31] Hampel, H. et al. The cholinergic system in the pathophysiology and treatment of Alzheimer’s disease. *Brain* **141**, 1917–1933 (2018). 29850777 10.1093/brain/awy132 PMC6022632

[32] Knezevic, E., Nenic, K., Milanovic, V. & Knezevic, N. N. The role of cortisol in chronic stress, neurodegenerative diseases, and psychological disorders. *Cells* **12**, 2726 (2023). 38067154 10.3390/cells12232726 PMC10706127

[33] Assem, Y., Adie, S., Tang, J. & Harris, I. A. The over-representation of significant p values in abstracts compared to corresponding full texts: a systematic review of surgical randomized trials. *Contemp. Clin. Trials Commun.* **7**, 194–199 (2017). 29696186 10.1016/j.conctc.2017.07.007 PMC5898552

[34] Himmelstein, D. S. & Baranzini, S. E. Heterogeneous network edge prediction: a data integration approach to prioritize disease-associated genes. *PLoS Comput. Biol.* **11**, e1004259 (2015). 26158728 10.1371/journal.pcbi.1004259 PMC4497619

[35] Su, C. et al. Biomedical discovery through the integrative biomedical knowledge hub (iBKH). *iScience* **26**, 106460 (2023). 37020958 10.1016/j.isci.2023.106460 PMC10068563

[36] Zheng, S. et al. PharmKG: a dedicated knowledge graph benchmark for bomedical data mining. *Brief. Bioinform.* **22**, bbaa344 (2021). 33341877 10.1093/bib/bbaa344

[37] Knox, C. et al. DrugBank 6.0: the DrugBank Knowledgebase for 2024. *Nucleic Acids Res* **52**, D1265–D1275 (2024). 37953279 10.1093/nar/gkad976 PMC10767804

[38] White, J. PubMed 2.0. *Med. Ref. Serv. Q.* **39**, 382–387 (2020). 33085945 10.1080/02763869.2020.1826228

[39] Harms, M. Medline. *Physiotherapy* **95**, 149–150 (2009). 19635332 10.1016/j.physio.2009.07.001

[40] Beekly, D. L. et al. The National Alzheimer’s coordinating center (NACC) database: the uniform data set. *Alzheimer Dis. Assoc. Disord.* **21**, 249–258 (2007). 17804958 10.1097/WAD.0b013e318142774e

[41] Luo, R. et al. BioGPT: Generative Pre-trained Transformer for Biomedical Text Generation and Mining. *Brief. Bioinform.* **23**, bbac409 (2022). 36156661 10.1093/bib/bbac409

[42] Djeddi, W. E., Hermi, K., Ben Yahia, S. & Diallo, G. Advancing drug–target interaction prediction: a comprehensive graph-based approach integrating knowledge graph embedding and ProtBert pretraining. *BMC Bioinformatics* **24**, 488 (2023). 38114937 10.1186/s12859-023-05593-6 PMC10731821

[43] OpenAI et al. GPT-4 Technical Report. Preprint at 10.48550/ARXIV.2303.08774 (2023).

[44] Cox, D. R. Regression models and life-tables. *J. R. Stat. Soc. Ser. B Stat. Methodol.* **34**, 187–202 (1972).

[45] Statistical Methods for Research Workers. *Nature* **131**, 383–383 (1933).

[46] Mickey, R. M. & Greenland, S. The impact of confounder selection criteria on effect estimation. *Am. J. Epidemiol.* **129**, 125–137 (1989). 2910056 10.1093/oxfordjournals.aje.a115101

[47] Bursac, Z., Gauss, C. H., Williams, D. K. & Hosmer, D. W. Purposeful selection of variables in logistic regression. *Source Code Biol. Med.* **3**, 17 (2008). 19087314 10.1186/1751-0473-3-17 PMC2633005

