# Supplementary material for: Bridging the computational-experimental gap: leveraging large language model to prioritize Alzheimer’s therapeutics based on comparison of learning models
Source: Npj Health Syst. 2026 Feb 26;3:20. doi: 10.1038/s44401-026-00074-3 (PMC12945682; doi:10.1038/s44401-026-00074-3)
Supplement: Supplementary file 1 — Supplementary information [file 44401_2026_74_MOESM1_ESM.pdf]

Supplementary Materials: Bridging the Computational-Experimental Gap: Leveraging Large Language Model to Prioritize Alzheimer's Therapeutics Based on Comparison of Learning Models

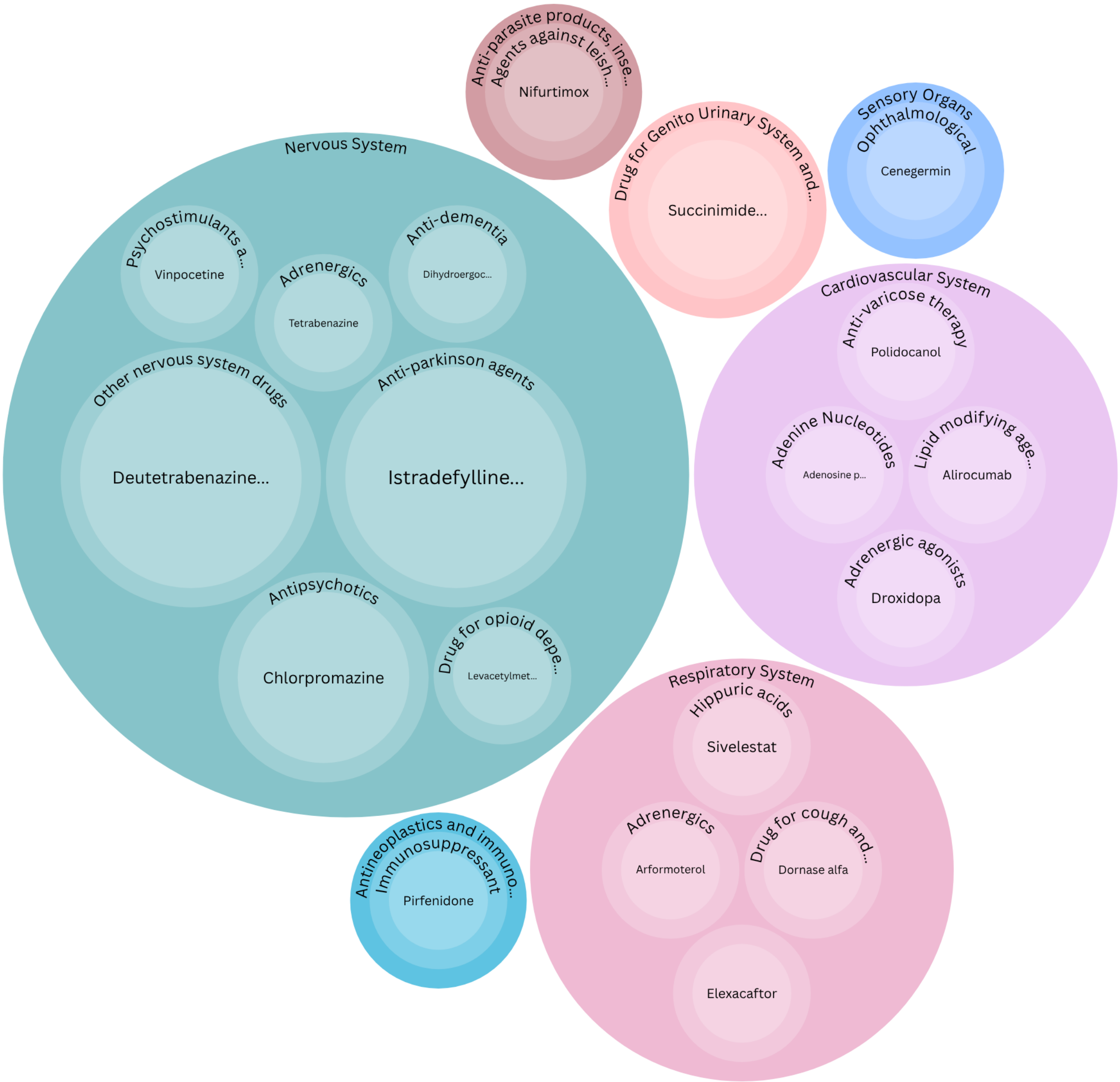

Supplementary Figure S1. ATC-classification landscape of the top 30 Alzheimer's disease candidates predicted by TxGNN.

This figure summarizes the Anatomical Therapeutic Chemical (ATC) Classification composition of the top 30 candidate drugs prioritized by TxGNN for Alzheimer's disease (AD). Drugs are grouped hierarchically by ATC categories: the outer, color-filled regions denote the ATC first-level anatomical main groups, and nested regions subdivide drugs into ATC therapeutic/pharmacological/chemical subgroups (lower ATC levels), with individual drug names shown within the corresponding ATC grouping. Across the top-30 list, the candidates span 19 ATC classes (mean 1.6 drugs per class), illustrating both breadth across anatomical systems and clustering within specific ATC categories among TxGNN-predicted therapeutics. **Abbreviations/Symbols/Color codes**: AD, Alzheimer's disease; ATC, Anatomical Therapeutic Chemical. Distinct fill colors indicate ATC first-level anatomical main groups as labeled in the figure (e.g., nervous system, cardiovascular system, respiratory system, genito-urinary system and sex hormones, sensory organs). Nested regions indicate lower-level ATC subgroupings; drug names are shown as text labels.

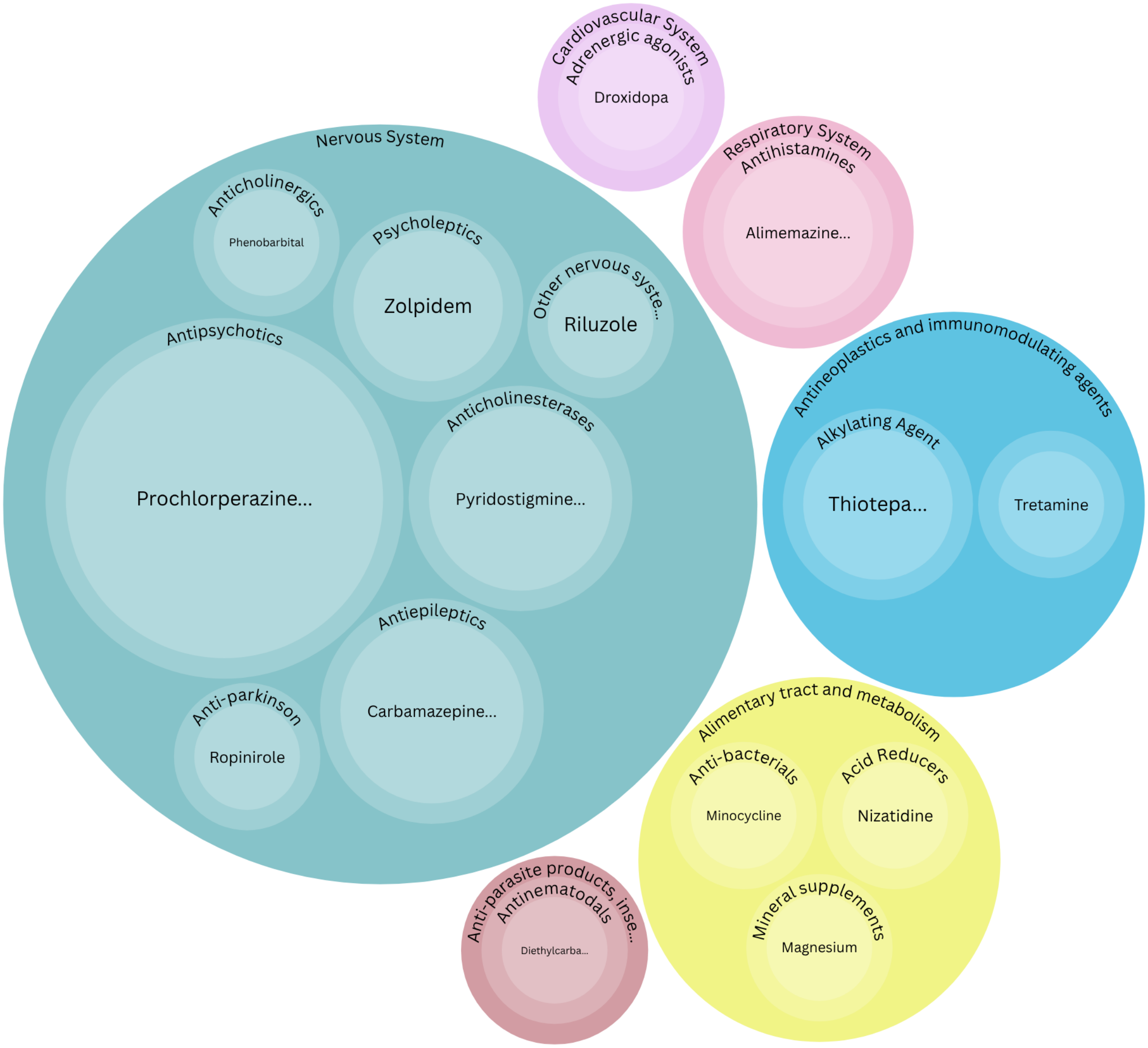

Supplementary Figure S2. ATC-classification landscape of the top 30 Alzheimer's disease candidates predicted by CompGCN.

This figure summarizes the Anatomical Therapeutic Chemical (ATC) Classification composition of the top 30 candidate drugs prioritized by CompGCN for Alzheimer's disease (AD). Drugs are grouped hierarchically by ATC categories: the outer, color-filled regions denote the ATC first-level anatomical main groups**,** and nested regions subdivide drugs into ATC therapeutic/pharmacological/chemical subgroups (lower ATC levels), with individual drug names shown within the corresponding ATC grouping. Across the top-30 list, the candidates span 15 ATC classes (mean 2.0 drugs per class), indicating that CompGCN concentrates predictions into fewer ATC categories than TxGNN while still covering multiple anatomical systems. **Abbreviations/Symbols/Color codes:** AD, Alzheimer's disease; ATC, Anatomical Therapeutic Chemical; CompGCN, compositional graph convolutional network. Distinct fill colors indicate ATC first-level anatomical main groups as labeled in the figure (e.g., nervous system, cardiovascular system, respiratory system, alimentary tract and metabolism, antineoplastic and immunomodulating agents). Nested regions indicate lower-level ATC subgroupings; drug names are shown as text labels.

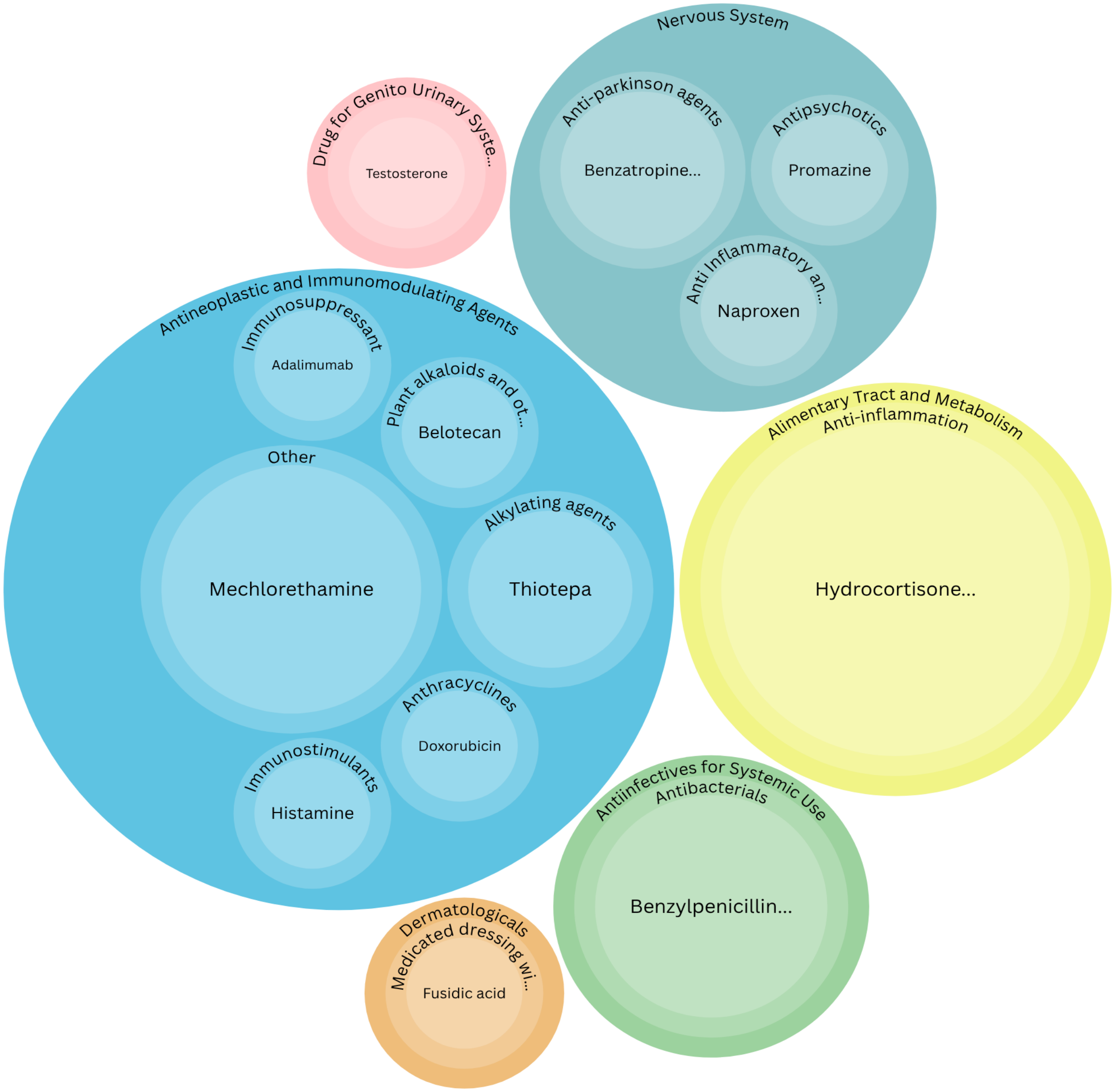

Supplementary Figure S3. ATC-classification landscape of the top 30 Alzheimer's disease candidates predicted by an RLR model using DWPC features.

This figure summarizes the Anatomical Therapeutic Chemical (ATC) Classification composition of the top 30 candidate drugs prioritized by a regularized logistic regression (RLR) model based on degree-weighted path count (DWPC) features for Alzheimer's disease (AD). Drugs are grouped hierarchically by ATC categories: the outer, color-filled regions denote the ATC first-level anatomical main groups, and nested regions subdivide drugs into ATC therapeutic/pharmacological/chemical subgroups (lower ATC levels), with individual drug names shown within the corresponding ATC grouping. Across the top-30 list, the candidates span 13 ATC classes (mean 2.2 drugs per class), reflecting a more concentrated ATC distribution for the DWPC-based RLR predictions. **Abbreviations/Symbols/Color codes:** AD, Alzheimer's disease; ATC, Anatomical Therapeutic Chemical (Classification); RLR, regularized logistic regression; DWPC, degree-weighted path count. Distinct fill colors indicate ATC first-level anatomical main groups as labeled in the figure (e.g., nervous system, alimentary tract and metabolism, antiinfectives for systemic use, dermatologicals, antineoplastic and immunomodulating agents). Nested regions indicate lower-level ATC subgroupings; drug names are shown as text labels.

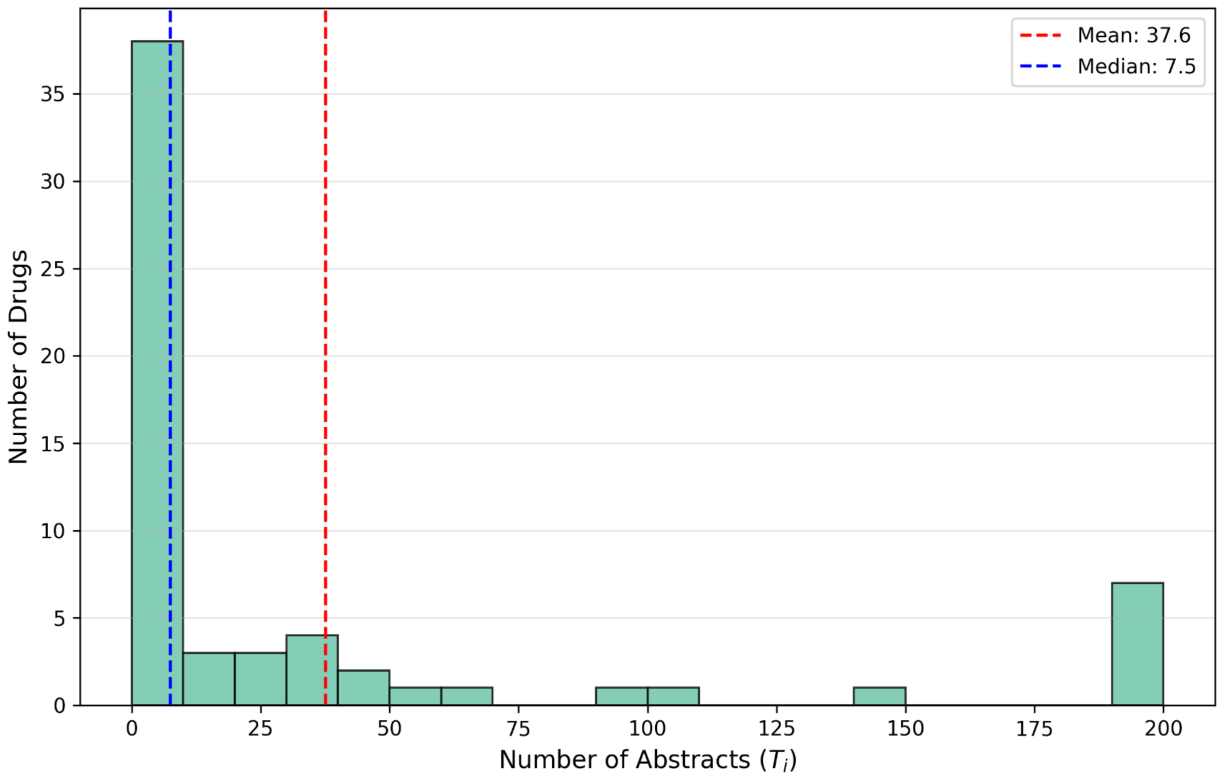

Supplementary Figure S4. Distribution of literature availability (T_i) across 62 candidate drugs.

The red dashed vertical line indicates the mean; the blue dashed vertical line indicates the median.

Supplementary Table S1. ATC classifications for the top 30 Alzheimer's disease candidates predicted by TxGNN.

Candidates are ranked in descending order based on predicted scores, reading from top to bottom, then left to right.

Drug Name	Chemical Family	Drug Name	Chemical Family	Drug Name	Chemical Family
Nifurtimox	Agents against leishmaniasis and trypanosomiasis (anti-parasite products, insecticides and repellents)	Opicapone	**Anti-parkinson agents (nervous system)**	Levacetylmethadol	**Drug for opioid dependence (nervous system)**
Deutetrabenazine	**Drugs for nervous system**	Cenegermin	Ophthalmological (sensory organs)	Dornase alfa	Drug for cough and cold preparations (respiratory system)
Levomethadone	**Drugs for nervous system**	Pimavanserin	**Antipsychotics (nervous system)**	Elexacaftor	Drugs in respiratory system
Tetrabenazine	**Adrenergics (nervous system)**	Vinpocetine	**Psychostimulants and nootropics (nervous system)**	Triflupromazine	**Antipsychotics (nervous system)**
Adenosine phosphate	Adenine Nucleotides (cardiovascular system)	Arformoterol	Adrenergics(respiratory system)	Dalfampridine	**Amines (nervous system)**
Istradefylline	**Anti-parkinson agents (nervous system)**	Alirocumab	Lipid modifying agents (cardiovascular system)	Tolcapone	**Anti-parkinson agents (nervous system)**
Piribedil	**Anti-parkinson agents (nervous system)**	Dihydroergocornine	**Drug for dementia and cognitive changes**	Clortermine	Amphetamines and derivatives
Safinamide	**Anti-parkinson agents (nervous system)**	Succinimide	Drug for genito urinary system and sex hormones	Pirfenidone	Immunosuppressant (antineoplastic and immunomodulating agents)
Valbenazine	**Drugs for nervous system**	Droxidopa	Adrenergic agonists (cardiovascular system)	Sivelestat	Hippuric acids (Drugs in respiratory system)
Chlorpromazine	**Antipsychotics (nervous system)**	Apomorphine	Drug for genito urinary system and sex hormones	Polidocanol	Anti-varicose therapy (cardiovascular system)

Supplementary Table S2. ATC classifications for the top 30 Alzheimer's disease candidates predicted by CompGCN.

Candidates are ranked in descending order based on predicted scores, reading from top to bottom, then left to right.

Drug Name	Chemical Family	Drug Name	Chemical Family	Drug Name	Chemical Family
Pyridostigmine	Anticholinesterases **(nervous system)**	Neostigmine	Anticholinesterases **(nervous system)**	Zolpidem	Psycholeptics **(nervous system)**
Prochlorperazine	Antipsychotics **(nervous system)**	Carbamazepine	Antiepileptics **(nervous system)**	Nizatidine	Acid Reducers (Alimentary tract and metabolism)
Tretamine	Antineoplastics and immunomodulating agents	Pimozide	Antipsychotics **(nervous system)**	Phenytoin	Antiepileptics **(nervous system)**
Phenobarbital	Antiepileptics; Anticholinergics **(nervous system)**	Memantine	Anti-dementia Drugs, Anticholinesterases **(nervous system)**	Acetophenazine	Antipsychotics **(nervous system)**
Thiotepa	Antineoplastics and immunomodulating agents	Paliperidone	Antipsychotics **(nervous system)**	Aprotinin	Antifibrinolytics (blood and blood forming organs)
Mechlorethamine	Antineoplastics and immunomodulating agents	Haloperidol	Antipsychotics **(nervous system)**	Perazine	Antipsychotics **(nervous system)**
Perphenazine	Antipsychotics **(nervous system)**	Procainamide	Antiarrhythmic (cardiovascular system)	Diethylcarbamazine	Antinematodals (anti-parasite products, insecticides and repellents)
Ropinirole	**Anti-parkinson agents (nervous system)**	Riluzole	**Other nervous system drugs**	Fluphenazine	Antipsychotics **(nervous system)**
Loxapine	Antipsychotics **(nervous system)**	Dextromethorphan	Antidepressive Agents Cough and cold preparations (**nervous system,** respiratory system)	Primidone	Antiepileptics **(nervous system)**
Alimemazine	Antihistamines (respiratory system)	Minocycline	Anti-infectives and antiseptics for local oral treatment; Anti-acne preparations; Anti-bacterials (Alimentary tract and metabolism; dermatologicals;antiinfectives for systemic use)	Magnesium	Mineral supplements (Alimentary tract and metabolism)

Supplementary Table S3. ATC classifications for the top 30 Alzheimer's disease candidates predicted by an RLR model using DWPC features.

Candidates are ranked in descending order based on predicted scores, reading from top to bottom, then left to right.

Drug Name	Chemical Family	Drug Name	Chemical Family	Drug Name	Chemical Family
Hydrocortisone	Corticosteroids; Anti-inflammation (alimentary tract and metabolism)	Thiotepa	Alkylating agents (antineoplastic and immunomodulating agents)	Testosterone	Drug for genito urinary system and sex hormones
Cortisone acetate	Corticosteroids; Anti-inflammation (alimentary tract and metabolism)	Fusidic acid	Medicated dressing with antiinfectives (dermatologicals)	Midostaurin	Antineoplastics
Hydrocortisone acetate	Corticosteroids; Anti-inflammation (alimentary tract and metabolism)	Carmustine	Alkylating agents (antineoplastic and immunomodulating agents)	Naproxen	Anti Inflammatory and antirheumatic products; Analgesics (nervous system, musculo-skeletal system,genito urinary system and sex hormones)
Dexamethasone	Corticosteroids; Anti-inflammation (alimentary tract and metabolism)	Belotecan	Plant alkaloids and other natural products (antineoplastic and immunomodulating agents)	Ofloxacin	Antibacterials (anti infectives)
Prednisolone	Corticosteroids; Anti-inflammation (alimentary tract and metabolism)	Benzylpenicillin	Antibacterials (antiinfectives)	Doxycycline	Anti-infectives and antiseptics for local oral treatment; Antibacterials (alimentary tract and metabolism, antiinfectives)
Doxorubicin	Anthracyclines (antineoplastic and immunomodulating agents)	Mechlorethamine	Antineoplastics	Adalimumab	Immunosuppressant (antineoplastic and immunomodulating agents)
Triamcinolone	Corticosteroids; Anti-inflammation (alimentary tract and metabolism)	Norfloxacin	Antibacterials (antiinfectives)	Histamine	Immunostimulants (antineoplastic and immunomodulating agents)
Betamethasone	Corticosteroids; Anti-inflammation (alimentary tract and metabolism)	Olaparib	Antineoplastics	Benzatropine	**Anti-parkinson agents (nervous system)**
Prednisone	Corticosteroids; Anti-inflammation (alimentary tract and metabolism)	Promazine	Antipsychotics **(nervous system)**	Procyclidine	**Anti-parkinson agents (nervous system)**
Methylprednisolone	Corticosteroids; (alimentary tract and metabolism)	Tretamine	Antineoplastics	Paclitaxel	Antineoplastics

Supplementary Table S4. Expert evaluation for 10 potential therapeutics.

Drug	Preclinical Effectiveness (0-4)		Safety and Tolerability (0-4)		Mechanism of Action (0-4)		Therapeutic Breadth (0-4)		Total (0-16)		Average (0-16)
Istradefylline	2	2	0	3	2	1	1	2	5	8	6.5
Chlorpromazine	2	0	1	4	2	1	1	1	6	6	6
**Pimavanserin**	2	3	3	3	2	2	1	2	8	10	**9**
**Droxidopa**	3	3	0	4	2	2	1	1	6	10	**8**
Apomorphine	2	3	0	3	1	2	1	1	4	9	6.5
Neostigmine	1	2	0	3	1	2	1	1	3	8	5.5
Carbamazepine	2	2	1	2	2	1	2	1	7	6	6.5
**Memantine**	3	3	4	4	4	3	2	3	13	13	**13**
**Riluzole**	2	3	2	3	2	2	1	2	7	10	**8.5**
**Minocycline**	3	3	2	3	2	2	1	2	8	10	**9**
**Magnesium**	3	3	0	4	3	1	2	2	8	10	**9**
Carmustine	2	2	1	1	2	1	2	1	7	5	6
**Testosterone**	2	1	1	3	2	2	2	3	7	9	**8**
**Doxycycline**	3	3	2	2	3	2	2	2	10	9	**9.5**
Histamine	1	0	0	2	2	1	0	1	3	4	3.5

Supplementary Table S5. Disagreement cases between experts and LLM analysis.

Drug_name	PubMed_ID	LLM results	Human
Benzylpenicillin	40028849	Negative	Neutral
Benzylpenicillin	39990831	Negative	Neutral
Benzylpenicillin	39953680	Negative	Neutral
Benzylpenicillin	26676721	Negative	Neutral
Carbamazepine	19488082	Neutral	Positive
Dextromethorphan	36075474	Negative	Neutral
Doxycycline	21998304	Neutral	Positive
Istradefylline	32357548	Neutral	Positive
Istradefylline	29100987	Neutral	Positive

Supplementary File S1. Evaluation Rubric for Assessing Alzheimer's Disease Drug Efficacy.

Please use the following rubric to quantitatively assess the efficacy of each drug listed for Alzheimer's Disease (AD) treatment based on your expertise. Rate each category **from 0 to 4**:

Scoring scale:

0: No evidence / Not effective
1: Minimal evidence / Slightly effective
2: Moderate evidence / Moderately effective
3: Strong evidence / Highly effective
4: Exceptional evidence / Extremely effective

Assessment Categories:

1. Preclinical Effectiveness

Improvement in cognitive function in animal models
Reduction in Alzheimer's pathology (e.g., amyloid plaques, tau phosphorylation)
Restoration of neurotransmitter or biochemical markers

2. Safety and Tolerability

Reported side effects or toxicity
Acceptability of safety profile in both animal and human studies

3. Mechanism of Action

Clear understanding of how the drug targets Alzheimer's pathology
Evidence supporting the proposed biological mechanism

4. Therapeutic Breadth

Drug's ability to address multiple aspects of Alzheimer's pathology (e.g., neuroinflammation, oxidative stress, amyloid clearance, cognitive symptoms)
